# Anti-aging effects of a functional food via the action of gut microbiota and metabolites in aging mice

**DOI:** 10.18632/aging.202873

**Published:** 2021-04-20

**Authors:** Jie Zhang, Zhewen Chen, Huaixi Yu, Yanwen Lu, Weinan Yu, Mingyong Miao, Hanping Shi

**Affiliations:** 1 Department of Endocrinology, The Affiliated Huai'an Hospital of Xuzhou Medical University, Huai’an 223002, Jiangsu, China; 2 Department of Nutrition, Zhejiang Provincial People's Hospital, Hangzhou 310000, Zhejiang, China; 3 Department of Orthopedics, The Affiliated Huai'an Hospital of Xuzhou Medical University, Huai'an 223002, Jiangsu, China; 4 Institute of BioPharmaceutical Research, Liaocheng University, Liaocheng 252059, Shandong, China; 5 Department of Biochemistry and Molecular Biology, The Naval Medical University, Shanghai 200433, China; 6 Department of Gastrointestinal Surgery, Department of Clinical Nutrition, Beijing Shijitan Hospital, Capital Medical University, Beijing 100038, China

**Keywords:** functional food, anti-aging, inflammation, immune function, gut microbiota and metabolites

## Abstract

Wushen (WS) is a mixed food containing 55 natural products that is beneficial to human health. This study aimed to reveal the preventive effect of WS on aging via a combined analysis of gut microbiome and metabolome. Senescence-accelerated mouse prone 8 (SAMP8) mice were used as aging model and senescence-accelerated mouse resistant 1 (SAMR1) mice as control. The mice were fed four diet types; control diet (for SAMR1 mice), standard diet (for SAMP8 mice, as SD group), WS diet, and fecal microbiota transplantation (FMT; transplanted from aging-WS mice). Our results showed that the weight, food intake, neurological function, and general physical conditions significantly improved in WS-fed mice compared to those fed with SD. The CA1 hippocampal region in WS-fed aged mice showed fewer shriveled neurons and increased neuronal layers compared to that of the SD group. WS-fed mice showed a decrease in malondialdehyde and an increase in superoxide dismutase levels in the brain; additionally, IL-6 and TNF-α levels significantly decreased, whereas IL-2 levels and the proportion of lymphocytes, CD3+CD8+ T, and CD4+IFNγ+T cells increased in WS-fed mice. After fed with WS, the abundance of *Ruminococcus* and *Butyrivibrio* markedly increased, whereas *Lachnoclostridium* and *Ruminiclostridium* significantly decreased in the aging mice. In addition, 887 differentially expressed metabolites were identified in fecal samples, among these, *Butyrivibrio* was positively correlated with D-glucuronic acid and *Ruminococcus* was positively associated with 5-acetamidovalerate. These findings provide mechanistic insight into the impact of WS on aging, and WS may be a valuable diet for preventing aging.

## INTRODUCTION

Aging is a progressive complex process that comprises a plethora of mechanisms such as senescence, immune-senescence and inflammation, representing important pathways of age-related diseases [1]. Estimates suggest that ~20% of the world population may be aged 65 or older by 2030, and have an increased prevalence of cardiovascular disease [2]. Diet is an important factor in aging, and individuals with a proper diet and nutrition management, including the consumption of antioxidant supplements, specific foods, and vitamins, may show a reduction in the rate of age-related diseases and a have a prolonged life span [3].

Natural products or nutraceuticals have been shown to elicit anti-aging, anti-cancer and other health-enhancing effects. Wushen (WS) is a food mixture composed of 55 different food ingredients which were rich in antioxidant, anti-inflammatory and immune-regulating substances. WS is composed of natural products without any additives and it is made from powders of fruits, vegetables, grains, aquatic products, cooked meats, eggs, and milk. Thus, WS can provide the human body with protein, fat and carbohydrates, and can supplement various minerals that human body lacks [4]. Many of the ingredients in WS, such as *Vitis vinifera* (Grape) and *Fagopyrum tataricum*, are reported to have anti-aging effects [5, 6]. Wang et al. revealed that WS had anti-tumor effect on S180 tumor-bearing mice and its mechanism was partly related to its antioxidant activity, suggesting that WS could be eaten directly and might be beneficial to human health [4]. However, the underlying mechanism of WS for anti-aging has not been elucidated.

WS contains many antioxidant and immune-related bioactive constituents such as crude polysaccharide, procyanidins, anthocyanin, resveratrol, squalene, vitamin C, selenium, and taurine. Antioxidants have the ability to delay aging and prevent age-related diseases through relieving oxidation [7]. For example, the crude polysaccharides extracted from *Calocybe indica* may reduce the occurrence of age-related diseases via their antioxidant activity [8]. In addition, resveratrol belongs to the family of natural phytoalexins and has been claimed as a master anti-aging agent against several age-associated diseases. Previous study has reported that gut microbiota is essential for regulating aging-related immunological, metabolic, and pathological pathways [9]. Thus, maintenance of healthy or young gut microbiota architecture may delay the aging process [10]. Procyanidin B2 is a component of natural plants or food and has the potential to prevent cognitive and oxidative impairment in d-gal induced aging in rats by regulating metabolic pathways and remodeling the gut flora [11]. Meanwhile, anthocyanin extracted from *Vaccinium myrtillus* L (Bilberries) could regulate the intestinal function of aging rats. After consumption Bilberry anthocyanin, bacteria beneficial (*Lactobacillus* and *Bacteroides*) to the intestine were inducted to grow, and harmful bacteria (*Verrucomicrobia* and *Euryarchaeota*) were inhibited [12]. All of these studies show that the active compounds of WS may be major contributors to changes in the composition of gut microbe, whereas none of them clarified the microbiota related to preventive effect of WS on aging.

More recently, fecal microbiota transplantation (FMT) has emerged as an effective therapy in preventing and treating age-related diseases, and FMT from wild-type mice contributes to the prolonged lifespan in progeroid mice [13]. Additionally, metabolic processes in the host are regulated by the gut microbiota [14]. For example, *Lactobacillus acidophilus* DDS-1 (a probiotic strain) can improve the metabolism of pathways associated with amino acids, proteins, and carbohydrates in aging mice [15]. In addition, Luo et al. [16] indicated that alteration of gut microbiota and metabolomics profiles were observed in the aging mice, and FuFang Zhenshu TiaoZhi might exert anti-aging effects by interfering with arachidonic acid metabolism, spingolipid metabolism, glycerolipid metabolism, and intestinal microbes of mice. Based on these studies, we intend to evaluate the preventive effect of WS on aging via integrated analysis of the changes in metabolites and gut microbiota.

Senescence accelerated mice-prone 8 (SAMP8) shows significant age-related deteriorations in memory and learning ability, which is consistent with the early onset and rapid advancement of senescence [17]. Normally, SAMP8 live 10 to 12 months on average and, at this time, they start to present a decline in learning and memory formation, and increased emotional disturbances (such as anxiety and depression), abnormal circadian rhythms, and brain atrophy [18]. Thus, SAMP8 mice have been used as a model for the study of brain aging and age-related neurodegenerative conditions. In this study, we aimed to investigate the effects of WS on metabolites and gut microbial composition of aging mice (SAMP8). We examined the neurological functions, inflammatory cytokines, antioxidant indicators, and the immune functions of SAMP8 mice, and further explored the changes in metabolites and gut microbiota.

## RESULTS

### WS improves the weight, food intake, and general physical conditions of aging mice

**Figure 1:**
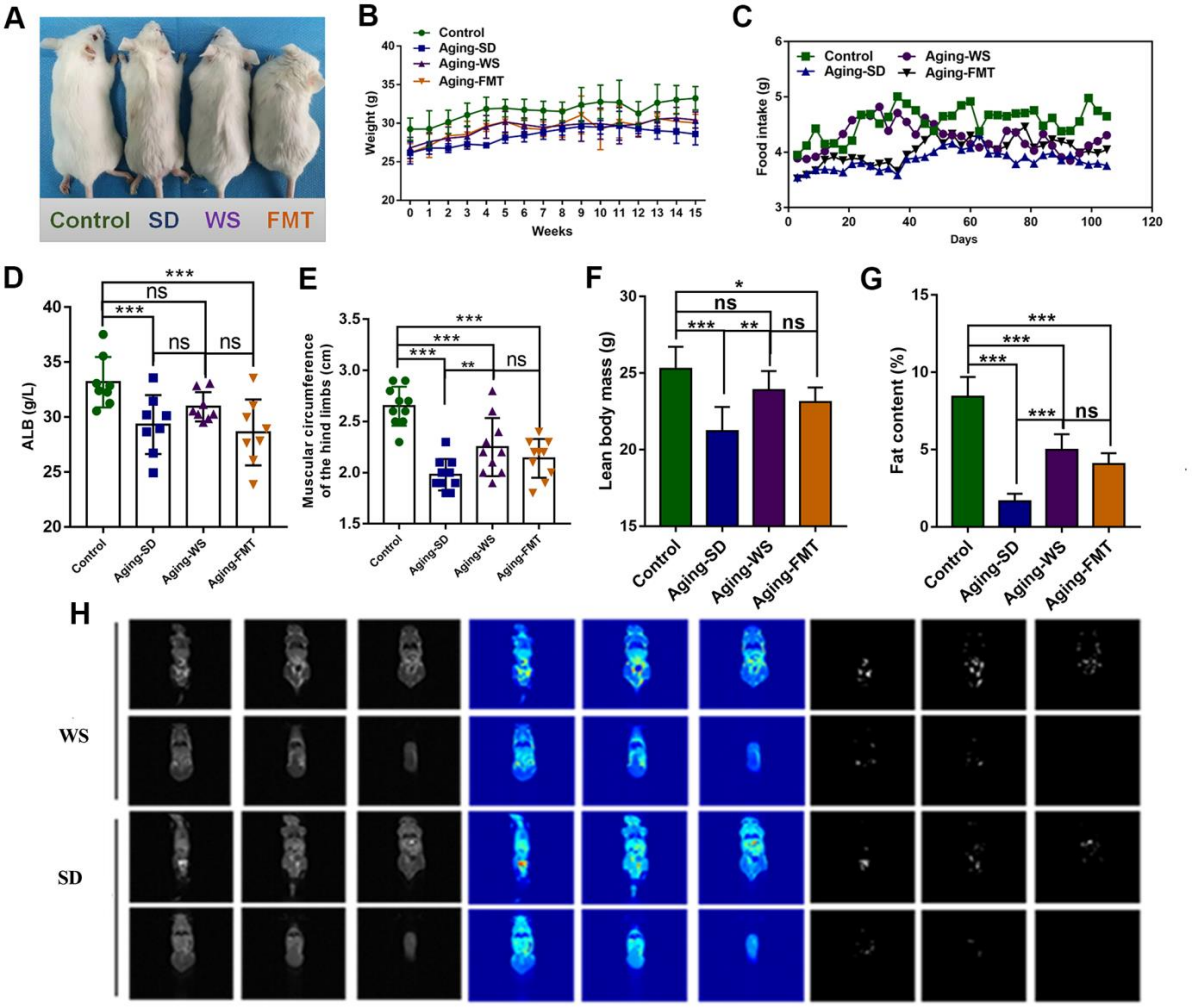
**The effects of WS on the general nutritional status of aging mice.** (**A**) The hair color and bodyweight of mice in each group. (**B**) The dynamic weight of mice in each group during the 0-15-week intervention process. (**C**) The food intake of mice in each group was recorded at days 0, 20, 40, 60, 80, 100, and 120. (**D**) The level of ALB (g/L) in the serum of mice. (**E**) The muscle circumference (cm) of the hind limbs of mice. (**F**) The lean body mass of mice. (**G**) The fat content in mice. (**H**) Analysis of body composition of mice. *P < 0.05, **P < 0.01, ***P < 0.001 in a comparison with the labeled group.

The hair color and body weight of the mice were recorded to note the base conditions during the dietary intervention process. As shown in Figure 1A, the hair color of the mice in the control group was snow-white, whereas the hair color of the aging mice in the SD, WS, and FMT groups was deep yellow. The mice in the SD group were thinner than the mice in the WS group. During the experimental intervention, the body weight and food intake of mice in the control group were found to be significantly higher than that of mice in the other three groups, and the administration of the WS and FMT resulted in a slight recovery in body weight (Figure 1B, 1C).

Further, the influence of WS on the physical fitness of the mice was examined. We found that the level of serum ALB was markedly decreased in the experimental group compared to the control group (*P* < 0.001), and the ALB levels were restored in mice from the WS group (Figure 1D). The measurements for the hind limb (Figure 1E) showed a distinct decrease in the muscle circumference in the SD group relative to the control group, whereas the administration of WS resulted in a significant increase in muscle circumference than in the SD group (*P* < 0.01). Compared to the control group, the lean body mass and fat content significantly decreased in the other three groups, while treatment with WS and FMT caused an increase in the lean body mass and fat percentage (Figure 1F, 1G). Magnetic resonance imaging was used to identify the fat distribution and content in mice from the SD and WS groups, and the subcutaneous fat content was lower in the SD group compared to the WS group (Figure 1H). These results suggested that WS might effectively alleviate the aging-induced reduction in food intake, body weight, serum ALB level, hind limb muscle circumference, lean body mass, and fat content.

### WS alleviates cognitive impairments and improves neurological function in SAMP8 mice

**Figure 2:**
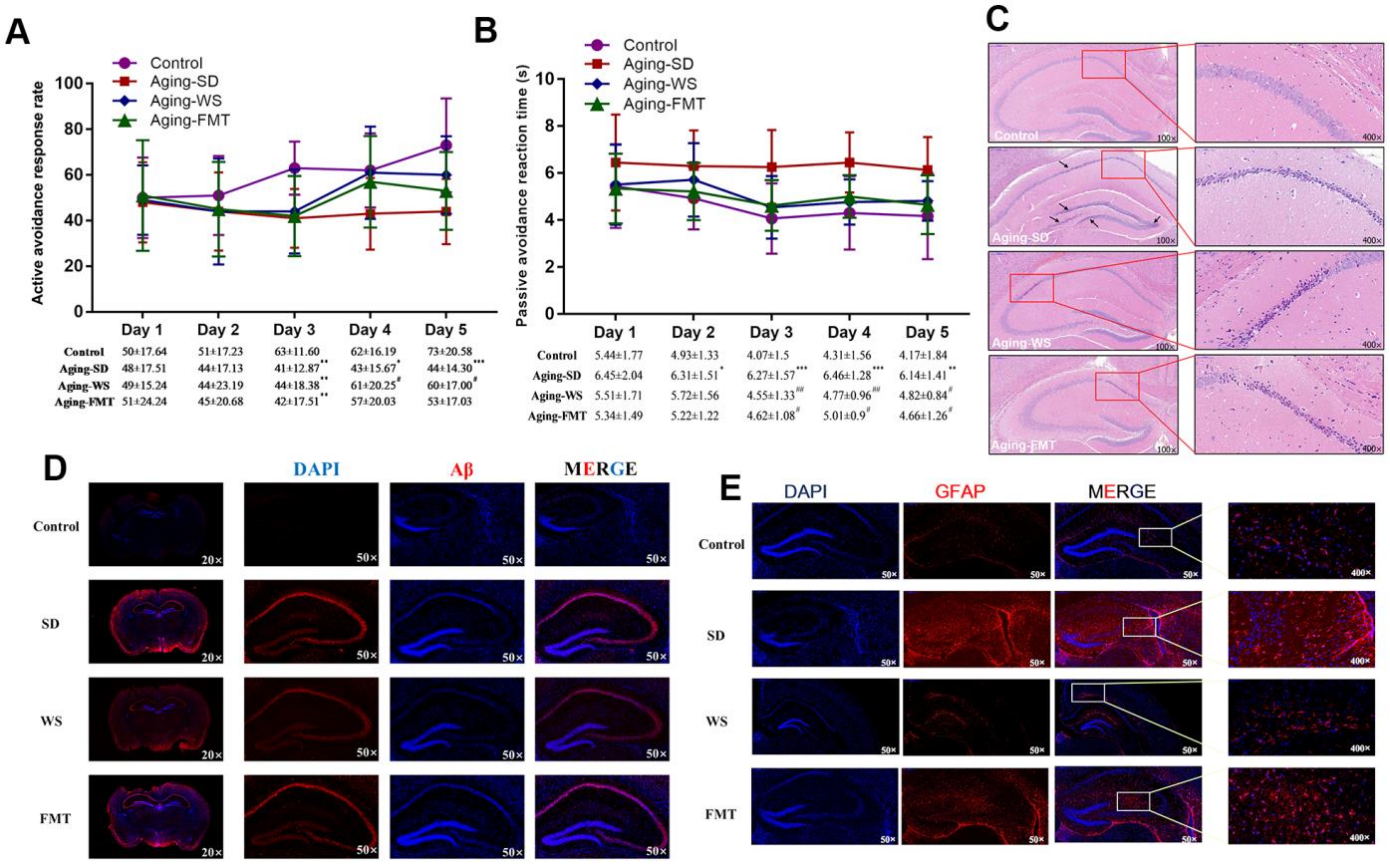
**Effects of WS on the learning memory and the neuronal molecules in mice.** (**A**) The ratio of active avoidance response (%). (**B**) Passive avoidance reaction time (s). *P < 0.05, **P < 0.01, ***P < 0.001 relative to the control group, and ^#^*P* < 0.05, ^##^*P* < 0.01, ^###^*P* < 0.001 relative to the SD group. (**C**) Images of the HE stained hippocampus in the mouse cerebrum at 100× and 400× magnification. (**D**) Immunohistochemical images of Aβ expression in tissues from the mouse cerebrum at 20× and 400× magnification. (**E**) Immunohistochemical images of GFAP expression in tissues from the mouse cerebrum at 50× and 400× magnification.

The learning and memorization abilities of mice were analyzed using the shuttle-box test. The results showed that the ratio of the successful avoidance response of mice in the control and WS groups was significantly higher than in the other experimental group from the 4^th^ day of training (Figure 2A, *P* < 0.05). The retention of the passive avoidance response of mice in the SD group was significantly higher than that in the WS and FMT groups from the 3^rd^ day of training (Figure 2B, P < 0.05). In addition, mice that were administered FMT and WS showed no significant difference compared with the control group. These results indicated that WS might ameliorate the learning and memorization abilities of SAMP8 mice.

The hematoxylin-eosin (HE) staining of the hippocampus (Figure 2C) showed that the features of cells in the CA1 region of the hippocampus in mice from the control group were abundant in neurons, and showed a normal morphological structure, clear cytoplasmic boundaries, and an evident nucleolus. However, the cells of mice in SD group were characterized by multiple shriveled neurons, reduced numbers of neurons and cell layers, nuclear hyperchromatism, unclear nucleolus, and rare cytoplasm. The cells from the CA1 region in the WS group showed deeper nuclear staining and no detectable shriveled neurons. The number of neuronal layers in WS group significantly increased relative to that in the SD group. Moreover, several shriveled neurons, a deeper nuclear staining, unclear cytoplasmic and nuclear boundaries, and relatively tightly packed neurons were observed in the cells of the CA2 region from the WS group. Notably, the characteristics of cells in the FMT group were similar to that in the WS group, except for the presence of a greater number of shriveled neurons. These results indicated that WS might improve the aging-induced morphological changes in the hippocampal cells in the cerebrum of mice.

Immunohistochemical (IHC) assays for the hippocampus showed that the expression of glial fibrillary acidic protein (GFAP, Figure 2E) and amyloid β-protein (Aβ, Figure 2D) considerably increased in mice from the SD group than those from the control group, while this effect was ameliorated in mice from the WS and FMT groups. These results demonstrated the effects of WS in the improvement of declining learning memory and neurological impairment in aging mice.

### Effects of WS on inflammatory cytokines, oxidation, and antioxidation indicators

The levels of inflammatory cytokines IL-2, IL-4, IL-6, IL-10, TNF-α, and IFN-γ in the serum of mice are shown in Table 1. The results indicated that the level of IL-2 in the control group was significantly higher than that in the SD, WS, and FMT groups (*P* < 0.001). In contrast, the levels of IL-6, IL-10, and TNF-α in the control group were significantly decreased relative to that in the SD, WS, and FMT groups (*P* < 0.001). Compared with the SD group, the IL-2 level significantly increased in the WS group (*P* < 0.001), whereas the IL-6 and TNF-α levels were obviously reduced in the WS group (*P* < 0.05). However, the levels of IL-2, IL-6, and TNF-α did not show significant differences between the WS and FMT groups. Further, no significant changes in the levels of IL-4 and IFN-γ were found among the four groups. These findings showed that WS might exert a partial anti-inflammatory effect by regulating several inflammation-related cytokines.

**Table 1:** Effect of WS on the inflammatory cytokines of mice.

**Parameters**	**Groups**			
	**Control**	**Aging-SD**	**Aging-WS**	**Aging-FMT**
IL-2	79.88±5.76	44.46±4.93***	66.19±7.92***###	58.64±10.06***
IL-4	51.92±9.56	43.01±4.37	49.61±7.75	46.84±9.29
IL-6	21.99±4.91	51.88±7.68***	44.36±4.01***#	50.57±7.15***
IL-10	21.84±2.19	37.26±6.82^***^	38.76±7.59^***^	36.93±1.82^***^
TNF-α	74.24±9.00	185.43±19.64***	153.97±23.56***##	146.19±18.51***
INF-γ	192.13±19.44	197.45±50.28	196.45±22.96	194.57±36.13

*P <0.05, *P < 0.01, ***P < 0.001 significant difference compared with control group; #*P* <0.05, ##*P* < 0.01, ###*P* < 0.001 significant difference compared with aging-SD group.

Additionally, the indicators for oxidation and antioxidation were also measured in the cerebrum of mice. The results demonstrated that the malondialdehyde (MDA) content in the cerebrum significantly decreased, and superoxide dismutase (SOD) and glutathione peroxidase (GSH-Px) was significantly higher in the control group than in the SD, WS, and FMT groups (Table 2, *P* < 0.001). Interestingly, the content of MDA in the cerebrum was significantly reduced in the WS group relative to the SD group (*P* < 0.001). Moreover, a significant activation of SOD activity in the cerebrum was detected in the WS group compared to the SD group. Notably, no significant differences of SOD and MDA activities were observed between the WS and FMT groups. Additionally, no significant change for GSH-Px activity was found in the SD, WS, and FMT groups. Together, these results demonstrated that WS had an antioxidative effect on aging mice.

**Table 2:** Effect of WS on the oxidation and antioxidation indicators of mice.

**Parameters**	**Groups**			
	**Control**	**Aging-SD**	**Aging-WS**	**Aging-FMT**
MDA	3.15±0.70	7.20±1.43***	5.45±0.47***###	6.03±0.88***
SOD	289.33±26.04	203.17±18.91***	230.61±21.29***#	211.69±16.81***
GSH-Px	87.04±9.50	39.69±8.95***	40.61±8.95***	40.68±8.69***

*P <0.05, *P < 0.01, ***P < 0.001 significant difference compared with control group; #*P* <0.05, ##*P* < 0.01, ###*P* < 0.001 significant difference compared with aging-SD group.

### WS improves immune functions in SAMP8 mice

We investigated the immune cell subsets in the spleen of the mice in the four groups. The results showed that the percentage of lymphocytes, natural killer (NK) cells, CD3^+^CD8^+^T cells, and CD4^+^IFNγ^+^T cells in the aging mice (SD, WS, and FMT groups) were significantly decreased relative to that of the control group. As expected, WS increased the number of these cells in the aging mice, and the percentages of these four cell types were not significantly different between the WS and FMT groups (Table 3). In addition, no significant change in the numbers of B cells, T cells, CD3^+^CD4^+^T cells, CD4^+^IL-4^+^T cells, and CD4^+^IL-17^+^T cells were detected among the four groups.

**Table 3:** Effect of WS on the immune cell subsets in the mouse spleen.

**Parameters**	**Groups**			
	**Control**	**Aging-SD**	**Aging-WS**	**Aging-FMT**
lymphocyte	62.09±6.51	51.03±6.39***	58.81±2.45##	51.59±3.64***&&
NK cells	2.10±0.24	1.23±0.23***	1.71±0.30**###	1.34±0.20***&&
B cells	51.35±4.09	49.23±5.23	49.04±3.49	51.73±1.32
T cells	38.01±3.95	36.90±3.49	39.03±2.79	37.09±1.95
CD3^+^CD4^+^ T cells	67.22±3.55	63.97±4.12	64.42±4.27	64.24±3.55
CD3^+^CD8^+^T cells	14.49±3.29	10.23±2.55**	14.42±3.35##	11.94±1.62
CD4^+^IFNγ^+^T cells	1.20±0.17	0.78±0.15***	0.95±0.12***##	0.91±0.14***
CD4^+^IL-4^+^T cells	0.74±0.09	0.72±0.11	0.68±0.07	0.65±0.87
CD4^+^IL-17^+^T cells	0.39±0.15	0.42±0.14	0.43±0.05	0.38±0.12

*P <0.05, *P < 0.01, ***P < 0.001 compared with control group; #*P* <0.05, ##*P* < 0.01, ###*P* < 0.001 compared with aging-SD group; &&*P* < 0.01 compared with aging-WS group.

### Influence of WS on bacterial diversity and composition in the gut

**Figure 3:**
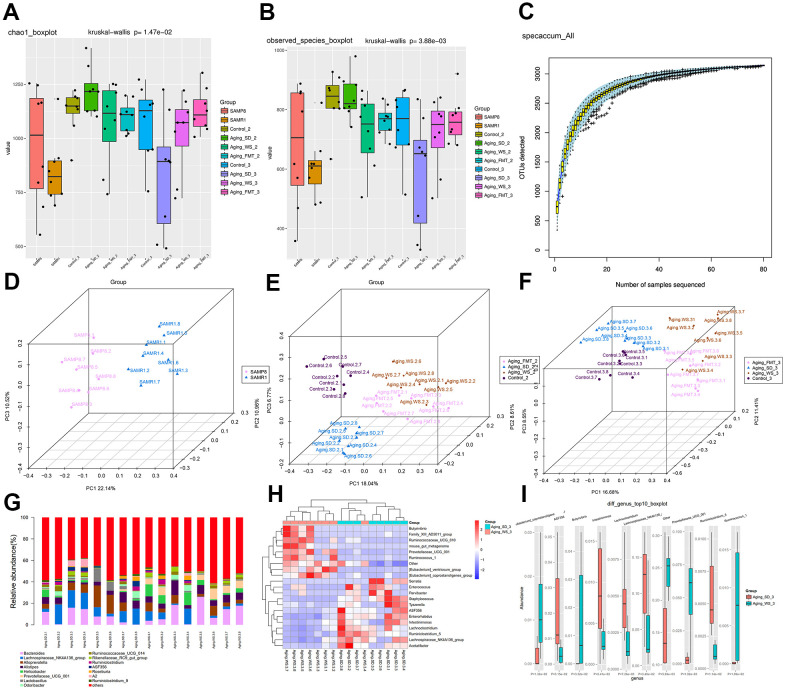
**The α- and β-diversity of gut microbiota from feces samples.** (**A**) Chao1 index of gut microbes in mice from the four groups. (**B**) The species index of gut microbes in mice from four groups. (**C**) Species accumulation curves. The X-axis represents the number of microbial samples sequenced, whereas the Y-axis represents the number of OTU detected. (**D**) PCoA plot showing the distribution of feces samples from SAMP8 and SAMR1 mice at the experiment baseline. (**E**) PCoA plot showing the distributions of plasma samples detected in four groups at the experiment’s metaphase. (**F**) PCoA plot showing the distributions of plasma samples from SAMP8 and SAMR1 mice at the experimental endpoint. The different colors represent samples from different groups. The closer the samples are, the more similar the microbial composition and structure between the samples. (**G**) The relative abundance of the top 15 differential OTUs between the WS and SD groups. (**H**) The heatmap for the top 20 differential OTUs. (**I**) The boxplot for the top 10 differential OTUs.

We profiled the gut microbiota in fecal samples from each mouse via 16S rRNA gene sequencing. The α-diversity (Chao1 index) analysis revealed that the types of bacterial species and their abundance in the gut microbiota from SD group were significantly decreased relative to mice from the other three groups (Figure 3A, 3B). No significant differences were observed in the gut microbiota among control, WS, and FMT groups, indicating the potential capability of WS to restore the altered imbalance of gut microbial distribution in the aging mice. Mice in the FMT group mimicked the status of intestinal microbiota from the WS group to some extent. In addition, the species accumulation curves demonstrated that the number of microbial species detected showed a leveling off, indicating that sufficient sampling had been achieved and our results were reliable (Figure 3C).

Furthermore, the β-diversity of the bacterial community in each group of mice during the baseline, metaphase, and end-point periods were compared. The principal coordinates analysis (PCoA) plots showed a distinct gut microbial community structure in a comparison between the control and SD groups, accounting for 43.62% of the total variability in the data (PC1=22.14%, PC2=10.96%, and PC3=10.52%; Figure 3D). After the administration of WS and FMT, the bacterial community structure in the group of aging mice that were administered SD were found to be increasingly similar to the control group as the intervention time increased (Figure 3E, 3F). In addition, we found that the bacterial community structure of FMT group was highly similar to that of WS group; however, these results were not consistent, indicating that the status of intestinal microbes in mice from the FMT group was simulated from the WS group to a certain extent.

Subsequently, the top 15 relatively abundant microbes in SD and WS groups were identified (Figure 3G), and the top 20 as well as top 10 differential genera are respectively presented in the heatmap (Figure 3H) and boxplot (Figure 3I). Results indicated that the abundance of bacteria from the family Prevotellaceae, and the genera *Ruminococcus* and *Butyrivibrio* markedly increased in the WS group relative to the SD group, whereas the abundance of *Lachnoclostridium*, *Ruminiclostridium*, *Eubacterium coprostanoligenes*, *Intestinimonas*, *Clostridium* sp. ASF356 and microbes from the family Lachnospiraceae significantly decreased in the WS group compared to the SD group.

### Comparison of the number of differential metabolites at different experimental times

The differences in the metabolites level between the SAMP8 and SAMR1 mice at baseline, 8 weeks, and 15 weeks were analyzed. PCA plot revealed that the distribution of metabolites in SAMP8 and SAMR1 mice was clearly separated at the baseline, indicating that the SAMP8 samples had distinct metabolic characteristics relative to the SAMR1 controls. However, the distance between the two sample types gradually approached during the experiment. In brief, the amount of the different metabolites between the SAMP8 and the SAMR1 mice significantly, decreased from 1131 at baseline, to 917 at 8 weeks, and to 111 at 15 weeks (end-point) (Supplementary Figure 1A). Further, no separate distributions for metabolites were observed between the SD and WS groups at 8 weeks; however, distinct distributions were presented at 15 weeks. Specifically, the number of differentially expressed metabolites between the SD and WS groups increased at 15 weeks (887) compared with 8 weeks (56) (Supplementary Figure 2B). Furthermore, for the samples from the WS and FMT groups, the PLS-DA plots showed an approaching distance at 15 weeks compared to 8 weeks, and the number of differentially expressed metabolites between the WS and FMT groups was reduced at 15 weeks (632) than at 8 weeks (375) (Supplementary Figure 2C). These results indicated that the FMT group simulated the alterations of metabolites from the WS group at 15 weeks. However, there was a certain difference in the number of metabolites between the WS and FMT groups, suggesting that the simple intestinal fecal transplantation in this study could not completely replicate the efficacy of oral WS.

### Pathways analysis of differentially produced metabolites between the WS and SD groups

**Figure 4:**
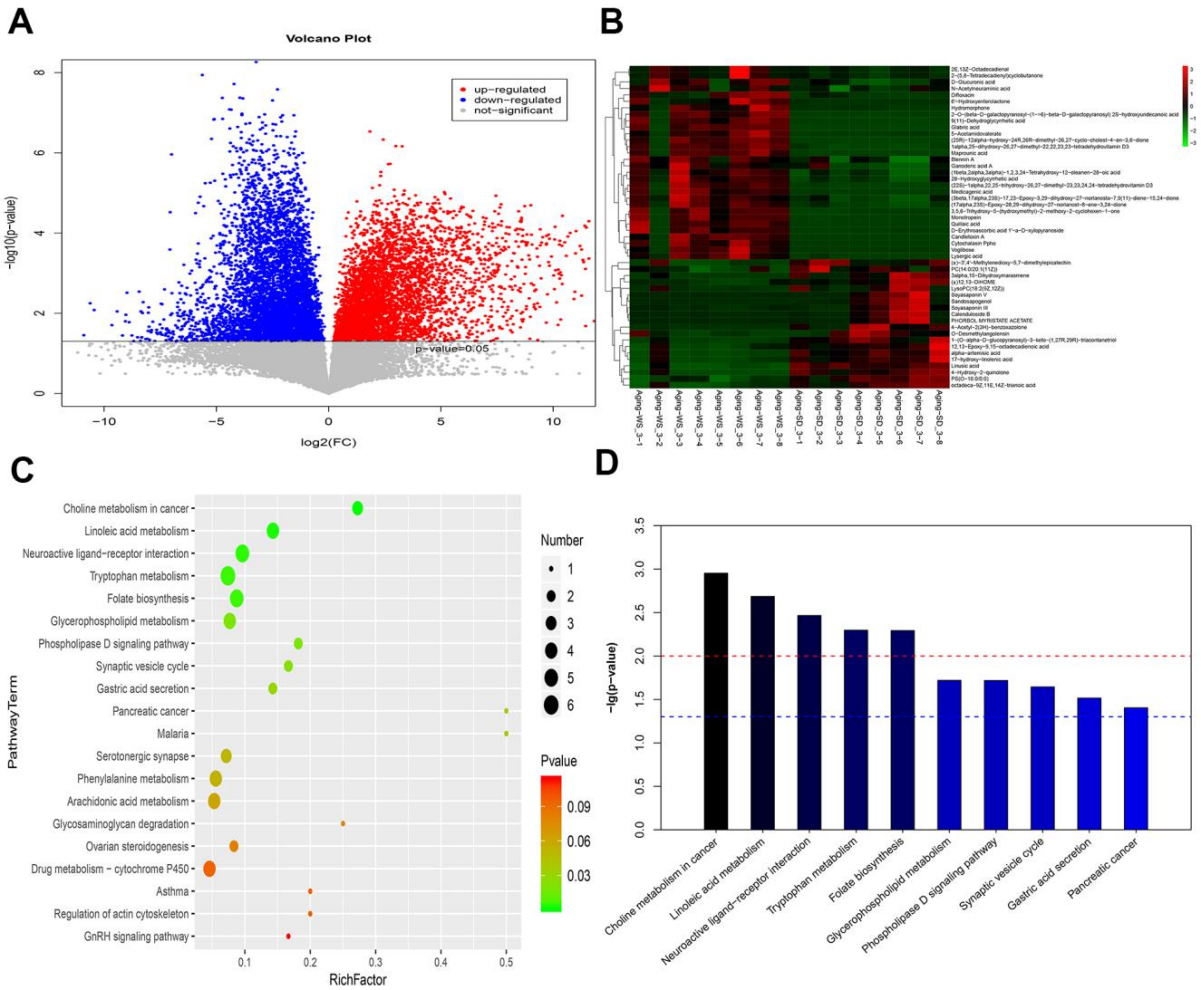
**The enriched functional pathways for the differential metabolites between the WS and SD groups from feces samples.** (**A**) The volcano plot for different metabolites. (**B**) The heatmap for the top 50 differentially enriched microbes. (**C**) The bubble map for the top 20 significantly enriched KEGG pathways. (**D**) The bar graph for the top 10 significantly enriched KEGG pathways.

For the fecal samples, compared with the SD group, 887 differentially expressed metabolites (eg., vitexin, melatonin, and histamine) were identified in the WS group. The volcano plot of the metabolites is shown in Figure 4A, 4B shows the heatmap for the top 50 differentially expressed metabolites. Based on the VIP values, the top metabolites including 2E, 13Z-octadecadienal, alpha-artemisic acid, and D-glucuronic acid were significantly upregulated in the WS group, whereas 4-Pyridoxic acid, 17-hydroxy-linolenic acid, 5-Hydroxyindoleacetic acid, and (S)-(-)-Perillyl alcohol were significantly downregulated. The log_2_ fold-change (FC) values indicated that Tetrahydrofolyl-[Glu](n), diltiazem, candletoxin A, formyl-5-hydroxykynurenamine, ponasterone A, quillaic acid, and vitexin were the top significantly upregulated metabolites in the WS group, whereas propinol adenylate, synaptolepis factor K1, and dehydrosoyasaponin I were the top significantly downregulated metabolites in the WS group (Supplementary Table 3). The results for the pathway enrichment analysis indicated that the differentially expressed metabolites were significantly involved in choline metabolism in cancer, linoleic acid metabolism, and neuroactive ligand-receptor interaction pathways (Figure 4C, 4D).

In addition, 121 differentially produced metabolites were screened from the serum samples of the WS and SD groups. The volcano plot and top 50 metabolites are shown in Supplementary Figure 2A, 2B. Based on the VIP values, the top three metabolites were PA(17:1(9Z)/22:2(13Z,16Z), LysoPC(18:1(11Z), and PC(14:0/22:4(7Z,10Z,13Z,16Z) (Supplementary Table 4). These differential metabolites were significantly associated with protein digestion and absorption, aminoacyl-tRNA biosynthesis, and glycerophospholipid metabolism pathways (Supplementary Figure 2C, 2D).

### Correlation analysis between microbial and metabolic profiles

**Figure 5:**
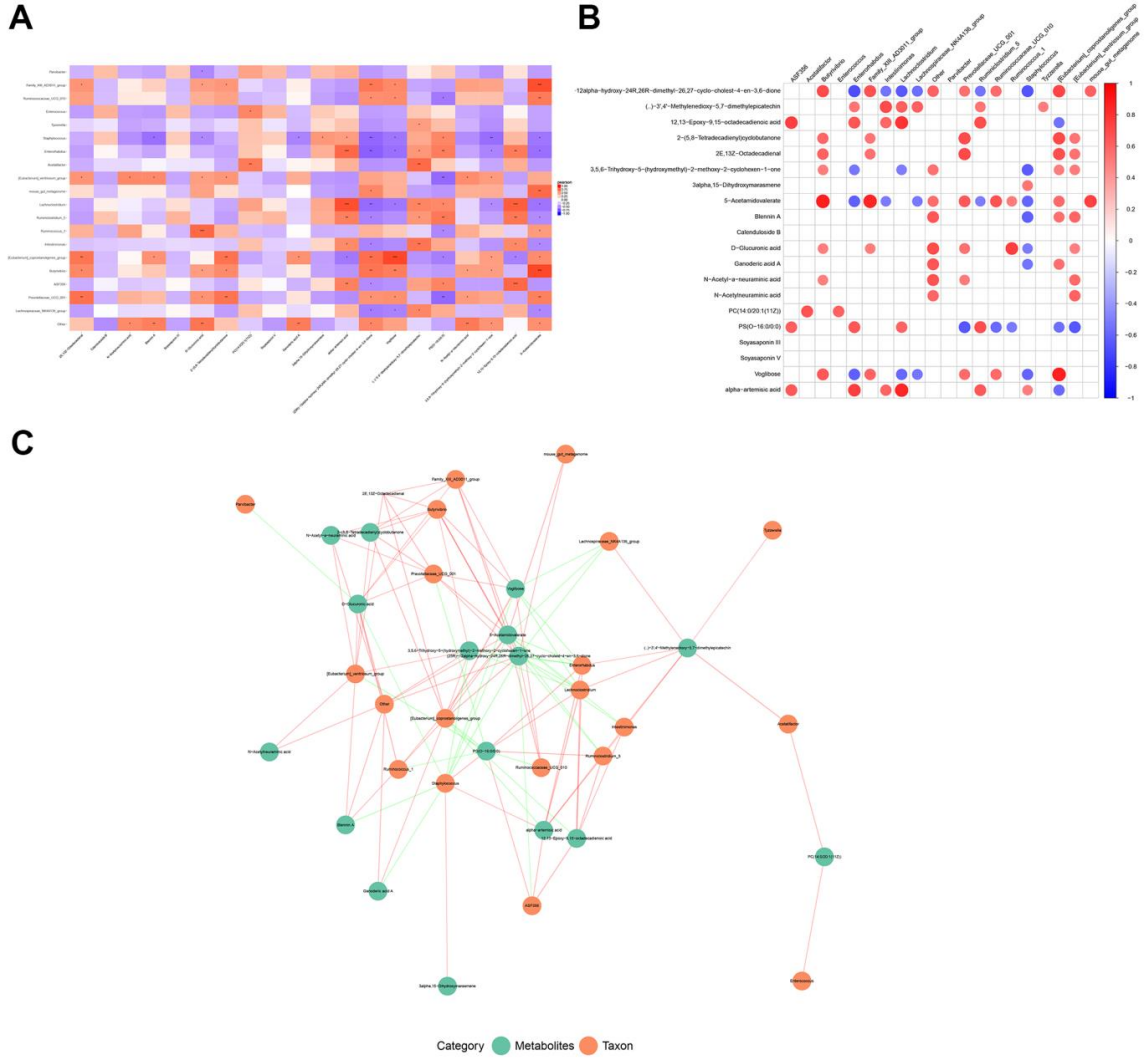
**The relationship between changed microbiotas and altered metabolites.** (**A**) Correlation square heatmap, (**B**) circular heatmap, and (**C**) the network of associations between the differential OTUs of gut microbes and the differentially expressed metabolites. Red color indicates a positive correlation and the blue color represents a negative correlation. A deeper color indicates a greater degree of correlation (*P < 0.05 and **P < 0.01).

Next, we explored the relationships between the differential microbes and altered metabolites based on Person’s correlation coefficient. A total of 535 microbe-metabolite relationship pairs involving 21 microbes and 94 metabolites were obtained. The heatmap and network of the top 20 correlation pairs are shown in Figure 5A–5C. The results showed that *Lactobacillus* positively correlated with calenduloside B (r=0.659, *P*=0.006), soyasaponin III (r=0.591, *P*=0.016), as well as soyasaponin IV (r=0.625, *P* =0.010), and negatively correlated with ganoderic acid A (r=-0.506, *P*=0.046); *Butyrivibrio* positively correlated with D-glucuronic acid (r=0.499, *P*=0.049), N-acetyl-a-neuraminic acid (r=0.498, *P*=0.049), and 5-acetamidovalerate (r=0.867, *P*<0.001); *Ruminococcus* positively correlated with D-glucuronic acid (r=0.745, *P*=0.001) and 5-acetamidovalerate (r=0.513, *P*=0.042).

## DISCUSSION

Aging is a complex and multifactorial process, which is accompanied by changes in the gut microbiota and metabolites. Diet represents the major extrinsic factor that influences the makeup and activity of resident intestinal microbes, thus the dietary intervention may be a beneficial method to improve intestinal flora disorders in the elderly [19]. WS, as a functional food containing various natural products, has been found to be beneficial to human health [4]. Our study revealed that WS was associated with a series of anti-aging events, including improved the cognitive impairments and neurological function of aging mice, reduced the level of several inflammatory and oxidative indicators, as well as increased the number of some immune cells. Moreover, the gut microbial and metabolic profiling analyses showed that the presence of several microbes and metabolites was altered in aging mice, and these changes could be restored using WS.

The decline in the learning memory ability is a typical feature of aging [20]. SAMP8 mice are models of spontaneously accelerated aging, showing exhibit neuropathological abnormalities and cognitive and behavioral alterations, such as deficits in learning and memory, oxidative stress, pathological changes in the cerebral cortex and hippocampus in the central nervous system, and alterations of immune function [21]. Thus, we explored the effects of WS on learning, neuropathological alterations and oxidative factors in aging mice. As observed in the shuttle-box test, the retention of the passive avoidance response of the SAMP8 mice was significantly prolonged and the memory of the animal also decreased. After fed with WS, the ability of learning and memory were improved, which indicated that WS effectively improved the age-relevant behavioral phenotype. Further, WS and FMT significantly alleviated the neuropathological alterations in SAMP8 mice such as neuronal loss and atrophy in the CA1 region of the hippocampus. We also found that WS and FMT greatly reduced the expression level of Aβ and GFAP in the hippocampus of aging mice. Aβ formation is thought to be one of the causes of neuronal and synaptic degeneration underlying cognitive decline in Alzheimer’s disease (AD). Previous study found that SS31 (a small molecule antioxidant peptide) could slow down cognitive decline of SAMP8 mice via lowering of central Aβ levels and protection of mitochondrial homeostasis [22]. Moreover, GFAP is a marker for the intermediate filament protein in astrocytes, which is the key glial cell type in the central nervous system [23, 24]. Reactive astrocytosis is a common feature of central nervous system (CNS) injury during the process of aging [25], and is often accompanied by increased GFAP expression [26]. Taken together, these results demonstrate the WS may serve the potential neuroprotective function in aging mice by decreasing the expression of Aβ and GFAP.

Aging is associated with chronic, low-grade increases in circulating levels of inflammatory marks [27]. Studies have shown that the testes in long-lived mice show anti-inflammatory and antioxidant capacities, whereas short-lived mice suffer from inflammatory and oxidative processes in the testes [28]. Ginés et al. [29] observed that the inflammatory status of old SMAP8 mice was elevated, and the protein expressions of TNF-α and IL-6 were increased, which was consistent with the results of our analysis. Notably, TNF-α and IL-6 are not only indicators of inflammation, but also causes of morbidity and mortality in the elderly [30]. In addition, we found that WS was able to decrease the pro-inflammatory status in SAMP8 mice, since the expression of TNF-α and IL-6 was significantly decreased after its administration. Aging is associated with pro-inflammatory cytokines, which might induce the formation of reactive oxygen species [31]. MDA is a marker of the oxidation index, whereas the SOD and GSH-PX indicators reflect the antioxidative properties of cells. Notably, it has been demonstrated that onjisaponin B can prevent cognitive impairment in d-gal induced aging in rats by regulating inflammatory mediators (TNF-α, IL-6, and IL-1β) and oxidative stress-related indicators (MDA, SOD, GSH, and GSH-PX) [32]. Similarly, our results indicated that the administration of WS significantly alleviated aging-related inflammation and oxidative damage by reducing the expression of MDA, IL-6, and TNF-α, and increasing the activity of SOD, GSH-PX. The antioxidant and anti-inflammatory activity of WS in aging rats may result due to its main components, of which the lutein [33], anthocyanin [34], zeaxanthin [35], and resveratrol [29] are well known antioxidative and anti-inflammatory molecules.

A crucial component of aging is a series of functional and structural alterations of the immune system that can manifest as a decreased ability to fight weaken immune response, autoimmunity and constitutive low-grade inflammation [36]. Previous study has reported that the features of an aged immune system include significantly reduced naïve T cell proliferation rate and changes in the subset composition of T cell caused by thymus shrinkage [37]. Abe et al [38] investigated the defects of immune cells in the senescence-accelerated mouse, and found that there were qualitative defects in CD4+T cells in SAMP8 mice, which might be closely related to the low endogenous activity of NK cells; these findings were consistent with the results of this study. Further, we observed that WS and FMT improved the immune functions in SAMP8 mice by enhancing the number of these immune cells.

In recent years, exciting advances have been made in the study of the mechanisms of aging, especially in work concerning gut microbiota, which is mainly altered by diet. In this study, we focused on altered microbial composition induced by aging and WS. Results revealed that the diversity and composition of the gut microbiome could be significant changed in aging mice, and WS and FMT had the potential to restore the imbalance of gut microbes in aging mice. Compared with SD group, diet with WS triggered marked changes in gut microbial composition, including an increase in *Ruminococcus* and *Butyrivibrio*, and a reduction in *Lachnoclostridium* and *Ruminiclostridium*. *Ruminococcus* is one of the key bacteria in the human colon microbiota, which is highly specific to resistant starch through the formation of amyloid [39]. It is also a microbial genus associated with age. Previous study showed that the abundance of *Ruminococcus* in the mice transplanted with fecal from long-living person was higher than that in the mice transplanted with fecal from elderly person [40]. *Butyrivibrio* is a butyrate-producing bacteria, and it has been confirmed to be connected with deterioration of clinical symptoms in health status. Luan et al. [41] observed that the abundance of Butyrivibrio showed significant reduction starting from 7 months before the death of the healthy centenarians. Moreover, butyrate can attenuate pro-inflammatory cytokine expression in microglia in aged mice, and can counterbalance the age-related microbiota dysbiosis, potentially improving neuro-inflammation [42]. These studies suggest that *Ruminococcus* and *Butyrivibrio* are beneficial bacteria that may help slow down the progress of aging. *Lachnoclostridium* and *Ruminiclostridium* have been found to be related to obesity, inflammation, and aging. However, the specific mechanism of their roles in the aging process has not been reported. Together, key gut microbiota related to aging had undergone a critical transformation due to WS intake effects.

Subsequently, we investigated the altered metabolites in SAMP8 mice fed with WS using LC-MS analysis. Compared with SD group, several upregulated metabolites such as vitexin and melatonin, and significantly downregulated metabolites such as histamine were detected in the WS group. The apigenin flavone glycoside vitexin possesses antioxidant and anti-inflammatory roles, and also has lifespan-extending activity [43]. Melatonin is known to reduce oxidative stress in aging cells [44]. In addition, the release of histamine that occurs in aging individuals may impact the aging process by the induction of an allergic reaction [45]. Taken together, we speculated that WS might alter the expression metabolites to play an anti-aging effect in SAMP8 mice.

To further explore the relationship between changed metabolites and altered microbiotas, we conducted a correlation analysis. Results revealed that *Ruminococcus* showed a significant positive correlation with the D-glucuronic acid. Studies have shown that *Ruminococcus gnavus,* as part of the gut microbiome, can produce inflammatory polysaccharides containing a rhamnose backbone and glucose sidechains to induce the secretion of TNF-α in dendritic cells [46]. Tang et al. identified an acid polysaccharide consisting of D-arabinose, D-xylose, D-glucose, D-galactose, D-galacturonic acid, and D-glucuronic acid, and demonstrated its antioxidant and anti-aging properties [47]. Further, we showed that WS increased the level of *Ruminococcus* and D-galacturonic acid, and reduced the expression of TNF-α, indicating that WS might exert an anti-aging effect via the activity of *Ruminococcus* to promote the secretion of D-glucuronic acid, thereby inhibiting inflammation factors such as TNF-α. In addition, we found that the presence of *Butyrivibrio* and 5-acetamidovalerate was positively correlated. However, the role of 5-acetamidovalerate in aging has not been clarified, thus further studies are required to explore this link in the development of aging. In this study, different types of metabolites have positive effects on the inflammatory response and gut microbial composition, so it is necessary to further analyze the correlation of hub metabolites and biochemical parameters.

To summarize, this is the first study to investigate the effect of WS on aging mice. Our findings demonstrated that dietary supplementation with WS could improve the symptoms of aging, including improved the ability of learning and memory, alleviated the neuropathological alterations, and enhances immune function. Moreover, compared with normal mice, the microbiota and metabolites of aged SAMP8 were significantly changed, while WS might restore them to normal levels. WS significantly increased the abundance of *Ruminococcus* and *Butyrivibrio*, and decreased the abundance of *Lachnoclostridium* and *Ruminiclostridium*. Moreover, correlation analysis distinctly revealed that *Butyrivibrio* was positively correlated with D-glucuronic acid and *Ruminococcus* was positively associated with 5-acetamidovalerate. Together, WS exerted anti-aging effects via modulating gut microbiota and metabolites, which could be valuable dietary prevention for aging.

## MATERIALS AND METHODS

### Preparation of WS

WS was produced by Yantai Wushen Food Science and Technology Co. Ltd. (Yantai, Shandong, China) using a series of special manufacturing processes, including spray drying, microwave treatment, and low-temperature baking. The proportions of protein, fat, carbohydrate, and fiber in WS were 22.1%, 12.7%, 36.36%, and 21.7%, respectively (Supplementary Table 1). In addition, WS is composed of 55 ingredients, and contains several antioxidant and immune-related nutrients such as crude polysaccharides (12,050 mg/100 g), squalene (164 mg/100 g), procyanidins (244 mg/100 g), anthocyanin (15.87 mg/100 g), vitamin C (24.4 mg/100 g), vitamin E (6.72 mg/100 g), zeaxanthin (236 mg/100 g), selenium (15.87 mg/100 g), and taurine (15.87 mg/100 g) (Supplementary Table 2).

### Animals and experimental design

All animal procedures were approved by the Institutional Animal Care and Committee of Second Military Medical University. A total of 60 male SAMP8 mice (aged 3-4 months) and 20 male senescence-accelerated mouse resistant 1 (SAMR1, aged 3-4 months) were purchased from Zhishan (Beijing) Institute of Health Medicine (Beijing, China). SAMP8 mice were allocated into three groups (n=20 per group): standard diet (SD) group, mice had ad libitum access to SD; WS group, mice had ad libitum access to processed WS laboratory feed; and FMT group, mice were fed with SD and were injected in the anus with 200 μL of fecal suspension derived from the WS group. SAMR1 mice fed with SD were used as control group. The SD consisted of 9% water, 19% protein, 4% fat, 5% fiber, 8% ash, and 55% carbohydrate. All mice were allowed to have free water, and maintained at standard temperatures (25 ± 2° C) and relative humidity (40 ± 5 %) conditions under 12 h light/dark cycle. The interventions were continued for 15 consecutive weeks.

### Shuttle-box test

Shuttle-box test was performed in accordance with a previous study [48], and it was used to evaluate the learning and memorization abilities of mice. Before the experiment, the mice were placed into chambers (25 ×18.5 ×30 cm) and acclimatized to the new situation for 5 min. Each training consisted of a conditioned stimulus (60 dB, 5 s) followed by an electrical shock (100 V, 50 Hz, AC, 10 s). If the mice escaped to the other side of the shuttle box before electrical stimulation, it is recorded as active avoidance. If the mice completed the shuttle after the electrical shock, it was considered as passive avoidance. Moreover, if no escape occurred after both stimuli, no avoidance behavior was recorded. The test was conducted for 5 consecutive days and 10 times per day with an inter-trial interval of 20 s. After the experiment, the response rates for active avoidance and passive avoidance were calculated to evaluate the memorization ability of mice. Additionally, the time needed for active and passive avoidance evaluated positively with the learning ability of the mice.

### Detection of basic indices

The body weight of each mouse was measured every week, and the daily food intake of mice was calculated. In addition, the activity and condition of the hair of the mice in each group were observed and recorded. At the end of the trial, the lean body mass and fat percentage of the mice in each group were examined by using the Awake animal body composition analyzer (MesoQMR23-060H; Shanghai Electronic Technology Co., Ltd, China). Meanwhile, the fat content and its distribution were detected by using the magnetic resonance imaging analyzer MesoMR23-060H-I.

### Sample collection

After the experiments, the mice were anesthetized using isoflurane and blood samples were collected. Serum was extracted after centrifugation of the blood samples at 3000 rpm for 15 min and stored at −80° C. Subsequently, the mice were sacrificed and immediately anatomized on ice to obtain the cerebrum, spleen, and hind limbs as described previously [4]. After washing with saline and drying with filter paper, the weights of the harvested organs were recorded. The muscle circumference of the hind limbs of the mice were measured and recorded. The whole hippocampus was isolated from the cerebrum for HE staining and IHC analysis. The feces (50 mg) of the mice at days 0, 8, and 15 weeks were collected and stored at −80° C for the analysis of short-chain fatty acids (SCFA) and the intestinal microflora.

### Hematoxylin-eosin staining and immunohistochemical analysis

The isolated hippocampus was immediately fixed upon isolation with 4% paraformaldehyde (Wuhan Servicebio Technology Co., Ltd., Wuhan, China), and subsequently dehydrated in ethanol and embedded in paraffin. Tissue sections of 5 μm thickness were prepared. For HE staining, the sections were stained with hematoxylin (Sigma-Aldrich) for 10 min, followed by incubation with eosin (Sigma-Aldrich) for 30 s at 25° C. The histological morphology of neurons in the CA1 area of the hippocampus was observed using an optical microscope (NIKON Eclipse Ci, Japan) at a magnification of 100× and 400×, respectively. For IHC staining, the sections were subjected to 3 min of microwave heating for antigen retrieval and were subsequently blocked with 5% goat serum (Sigma-Aldrich) at 37° C for 1 h. Next, the sections were incubated with rabbit anti-glial fibrillary acidic protein (GFAP; 1:500; Abcam) primary antibody at 4° C overnight, followed by treatment with horseradish peroxidase (HRP)-conjugated sheep anti-rabbit IgG (1:1500; Abcam) at 37° C for 1 h. The visualization of the immunoreaction was performed using 3, 3′-diaminobenzidine (Sigma-Aldrich). The stained images were photographed using a microscope at 400× magnification.

### Quantification of immune cells via flow cytometric analysis

Flow cytometry was used to identify the immune cell subsets from the fresh spleen tissues of mice. Briefly, the spleen tissues were dissociated into single cells by trituration and cell lysis with red blood cell lysis buffer (Cat. No: 420301; Biolegend, San Diego, CA, USA). After centrifugation, the density of the single-cell suspension was adjusted to 1 × 10^6^ cells/mL. The CD3^+^CD8^+^T cells, CD3^+^CD4^+^T cells, CD4^+^IFNγ^+^T cells, and Treg cells were stained with antibodies for labeling. Next, the percentage of the different immune cell subsets was identified by flow cytometry (FACSFortessa X20). The antibodies used were as follows: CD3 eF450 (Cat. No: 11-0042-82, Thermo), CD4 PE-Cy7 (Cat. No: 100528; BD Biosciences, San Jose, CA, USA), CD8a PerCP-Cy5.5 (Cat. No: 45-0081-82; Thermo), CD4 FITC (Cat. No: 11-0042-82; Thermo), and IFN eF450 (Cat. No: 48-7311-82; Thermo).

### Enzyme-linked immunosorbent assay (ELISA) analysis

The levels of inflammatory cytokines IL-2 (Cat. No: SU-B20010), IL-4 (Cat. No: SU-B20011), IL-6 (Cat. No: SU-B20012), IL-10 (Cat. No: SU-B20005), TNF-α (Cat. No: SU-B20220), and IFN-γ (Cat. No: SU-B20707) in the mouse serum were detected using ELISA kits from Kenuodi Biotechnology Co., Ltd (Fujian, China) as per manufacturers’ instructions. The oxidation marker MDA, and the antioxidant activities of SOD and GSH-Px in cerebrum samples were detected using ELISA kits from Sigma. Serum albumin (ALB) was detected using a kit from the Nanjing Jiancheng Bioengineering Institute (Nanjing, China).

### 16S rRNA sequencing

Total microbial genomic DNA (gDNA) from the fecal samples (5 g) of each mouse was extracted using the QIAamp DNA Stool Mini Kit (50) (51504, Qiagen) as per the manufacturer’s protocol. The V3-V4 regions of the bacterial 16S rRNA gene were amplified using the primers pairs 356F (5'-CCTACGGGNGGCWGCAG-3') and 803R (5'-GACTACHVGGGTATCTA ATCC-3') [49]. Next, the PCR amplification, the library preparation and sequencing were performed as described previously [49]. The Illumina Miseq (Illumina, San Diego, CA, USA) sequencing platform was used to generate paired-end sequencing reads (2×300 bp). Subsequently, the FLASH software (Trimmomatic) was used to merge the matched pairs and perform quality control for the reads obtained.

### Bioinformatics analysis

USEARCH (version 7.0) was used for Operational Taxonomic Units (OTU) clustering at a similarity level of 97%. The Ribosomal Database Project classifier v2.2 was applied to perform the taxonomy for each representative OTU against the Silva database [50]. Then, the obtained OTU data were used for taxonomical assignments using RDP Classifier algorithm (http://rdp.cme.msu.edu/classifier/classifier.jsp). The analyses for the richness index, alpha (Chao index [51]), and beta diversity (PCoA) were conducted through QIIME (Version 1.9, http://qiime.sourceforge.net/). The *t*-test and the Wilcoxon sum-rank test were used to assess the difference between the groups. The OTUs with *P*<0.05 were considered to be significantly different between groups. A principal component analysis (PCA) and UniFrac-based PCoA were performed using the R statistical software to perform the clustering of different samples.

### Metabolomic analysis

A total of 100 μL serum and 60 mg stool samples from each mouse in different groups (n=8) were collected. Subsequently, 10 μL and 20 μL of the internal standard (0.3 mg/mL L-2-chlorophenyl alanine and 0.01 mg/mL 17:0 Lyso PC) was added to the serum and stool samples, respectively, and 400 μL mixture of methanol and water (V:V=1:4) was added to remove impurities. The supernatant of each sample was used for liquid chromatography-mass spectrometry (LC-MS) analysis using Dionex UltiMate 3000 UHPLC, (Thermo Scientific). Chromatographic separation was conducted using a 100 × 2.1 mm, 1.8 μm ACQUITY UPLC HSS T3 column at 50° C with a flow rate of 0.35 mL/min. Water with 0.1% formic acid was used as the solvent A of the mobile phase, and acetonitrile with 0.1% formic acid was used as solvent B.

### Data pre-processing, multivariate analysis, and metabolic annotation

The Progenesis QI v2.3 software was used for data processing, including baseline filtering, peak identification, integration, correction of retention time, peak alignment, and normalization. Next, multivariate statistical analyses including PCA and orthogonal partial least-squares discriminant analysis (OPLS-DA) were used to observe the distribution of samples and the differential metabolites among groups. The degree of difference between the two groups was evaluated using the *t*-test, and the metabolites showing significant differences were selected using thresholds of VIP > 1 (VIP = variable importance in the projection) and *P*-values < 0.05. Volcano plots were created to identify the *P* and the fold-change values for metabolites between the groups. Next, hierarchical clustering was performed to identify the expression differences of the top 50 metabolites in different samples. The functions of differential metabolites were annotated using the Kyoto Encyclopedia of Genes and Genomes (KEGG) pathway analysis.

### Correlation analysis for microbial taxonomy and metabolites

To evaluate the potential associations between the different microbes and metabolites identified in the WS and SD groups, a Spearman’s correlation analysis was conducted. The Spearman’s correlation coefficient was calculated for the relative abundance of OTU and the response intensity of the metabolites, and the relationship between microbial taxonomy and metabolites. A network showing the top 20 results was plotted.

### Statistical analysis

Data were expressed as mean ± standard deviation (mean ± SD) and were analyzed using the SPSS 19.0 software (IBM, New York, NY, USA). Data from more than three groups were analyzed using a one-way analysis of variance (ANOVA) and the least significant difference (LSD) test. A value of *P* < 0.05 was considered as statistical significant.

## Supplementary Material

Supplementary Figures

Supplementary Tables 1 and 2

Supplementary Table 3

Supplementary Table 4
