# Supplementary material for: Anti-aging effects of a functional food via the action of gut microbiota and metabolites in aging mice
Source: Aging (Albany NY). 2021 Apr 20;13(13):17880–900. doi: 10.18632/aging.202873 (PMC8312451; doi:10.18632/aging.202873)
Supplement: Supplementary Figures [file aging-13-202873-s001.pdf]

SUPPLEMENTARY FIGURES

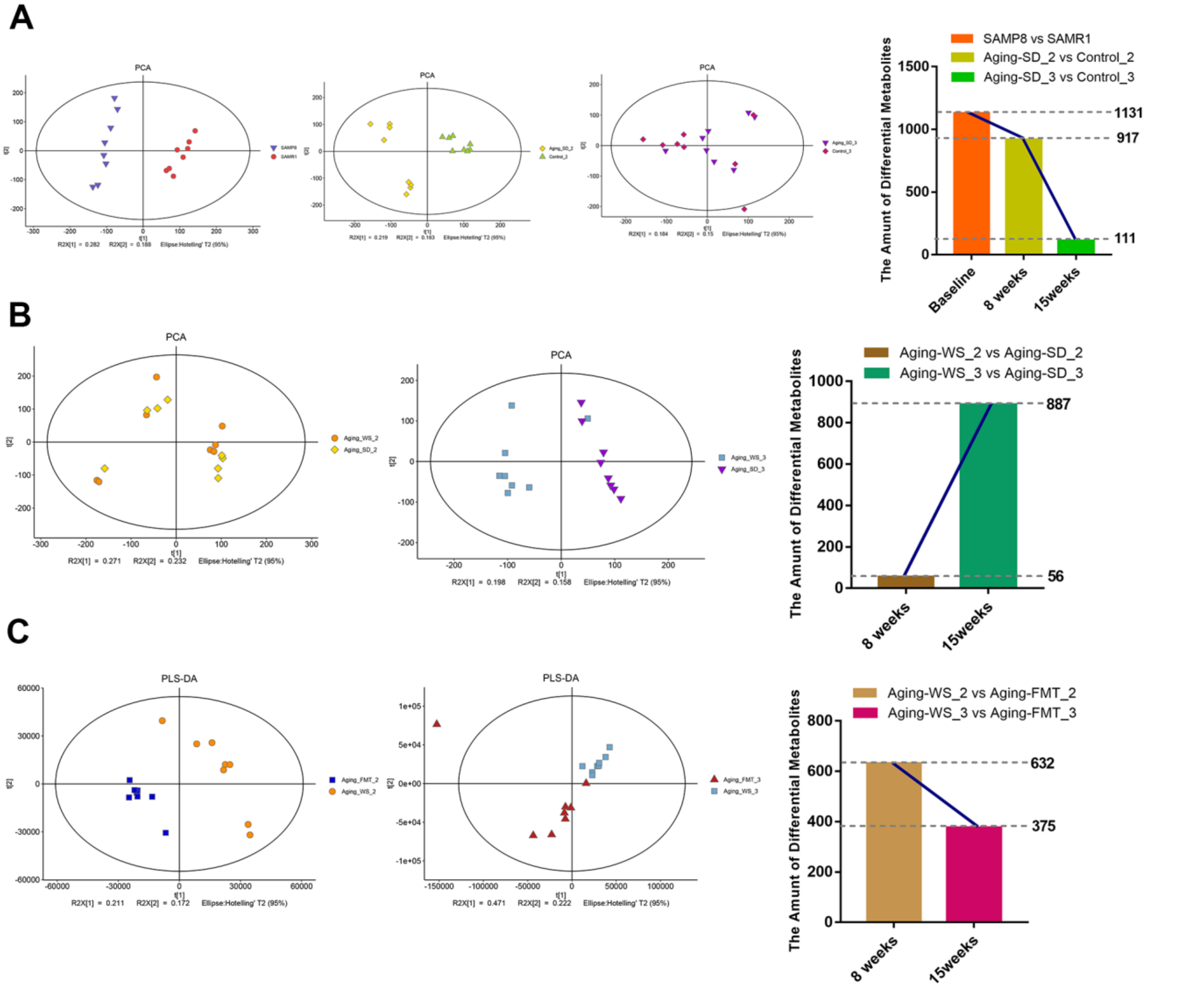

Supplementary Figure 1. The comparisons of numbers of differential metabolites at different experiment time from faeces samples. (**A**) PCoA plot and the counts of differential metabolites identified at baseline, 8w and 15w between SMPA8 and SMMR1 sample. (**B**) PCoA plot and the counts of differential metabolites identified at experiment 8w and 15w between SD and WS groups. (**C**) PCoA plot and the counts of differential metabolites identified at 8w and 15w between FMT and WS groups.

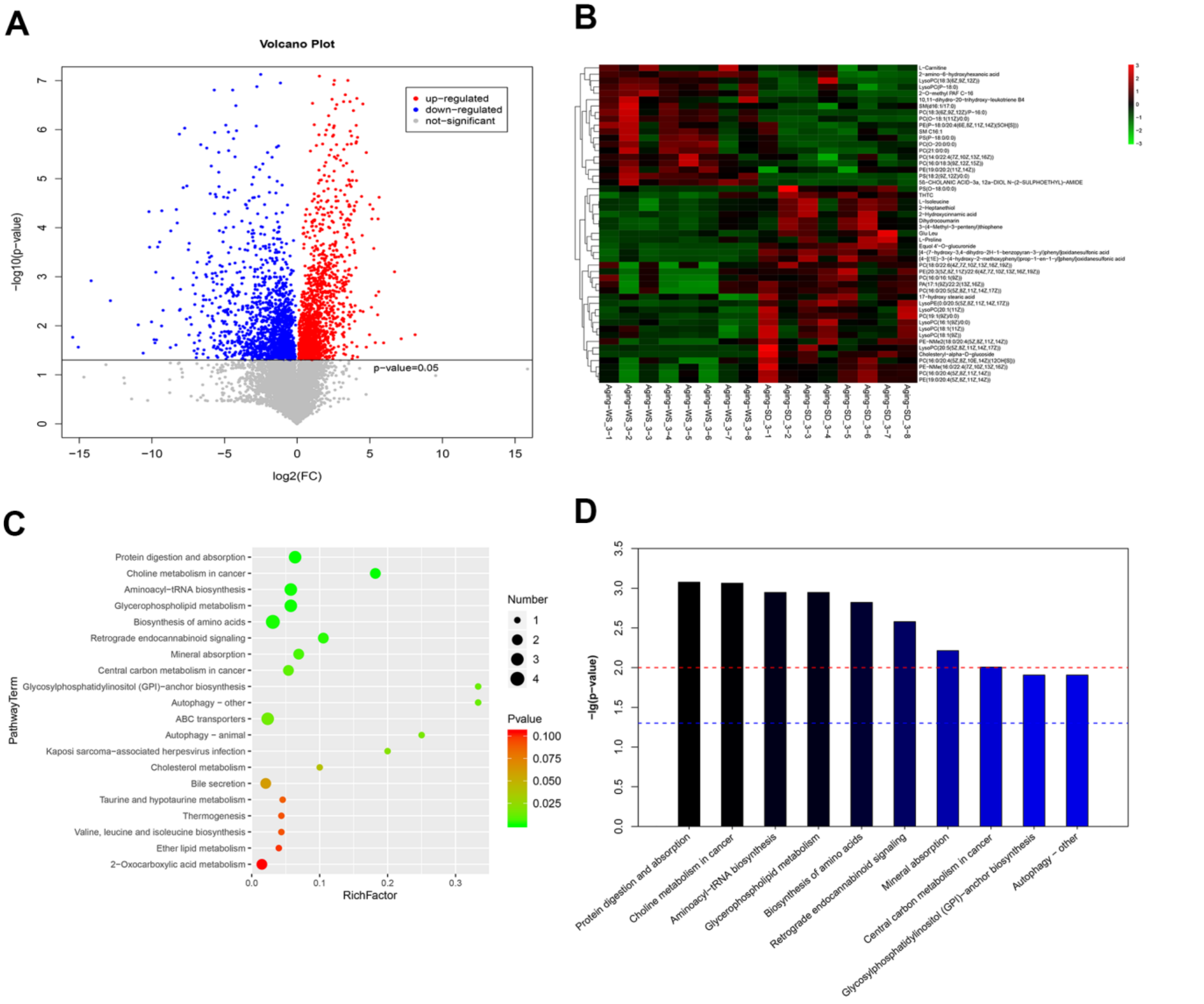

Supplementary Figure 2. The enriched functional pathways of differential metabolites between WS and SD groups from serum samples. (**A**) The volcano plot of differential metabolites. (**B**) The heatmap of top 50 differential microbials. (**C**) The bubble map of top 20 significantly enriched KEGG pathways. (**D**) The bar graph of top 10 significantly enriched KEGG pathways.
