# Supplementary material for: Anti-aging effects of a functional food via the action of gut microbiota and metabolites in aging mice
Source: Aging (Albany NY). 2021 Apr 20;13(13):17880–900. doi: 10.18632/aging.202873 (PMC8312451; doi:10.18632/aging.202873)
Supplement: Supplementary Tables 1 and 2 [file aging-13-202873-s002.pdf]

SUPPLEMENTARY TABLES

Supplementary Table 1. The ingredient of Wushen.

	Standard diet (%)	Wushen (%)
Protein	19	22.1
Fat	4	12.7
Carbohydrate	50	36.36
Fiber	5	21.7
Ash content	8	3.6
Water content	<10.0	3.49
Calories per gram	3.12kcal	3.91kcal

Supplementary Table 2. The main antioxidant and immune-related ingredient of Wushen.

Antioxidant components	Content (mg/100g)	Immunoenhancing components	Content (mg/100g)
Preanthocyanin,	244	Crude polysaccharides	12050
Anthocyanin	15.87	Squalene	1.64
Vitamin c	24.4	Selenium	16.9
Taurine	9.39	Taurine,	9.39
Vitamin E	6.72	Beta carotene	1.47
Lutein	1.78	Vitamin A	0.007
Squalene	1.64	Total flavonoids	0.1%
Zeaxanthin	236		
Resveratrol	<0.3		
Total flavonoids	0.1%		
