# Supplementary material for: Anti-aging effects of a functional food via the action of gut microbiota and metabolites in aging mice
Source: Aging (Albany NY). 2021 Apr 20;13(13):17880–900. doi: 10.18632/aging.202873 (PMC8312451; doi:10.18632/aging.202873)
Supplement: Supplementary Table 3 [file aging-13-202873-s003.docx]

**Supplementary Table 3. The top 50 differential metabolites based on VIP values and log2(FC) values identified between WS and SD groups from faeces samples.**

| **Metabolites** | **Compound ID** | **Formula** | **VIP** | **adj.P-value** | **log2(FC)** |
| --- | --- | --- | --- | --- | --- |
| **Top 50 metabolites based VIP values** |  |  |  |  |  |
| 2E,13Z-Octadecadienal | LMFA06000234 | C18H32O | 22.586453 | 0.1842178 | 1.557909 |
| Calenduloside B | HMDB0039413 | C48H78O18 | 15.997019 | 0.1737437 | -4.662279 |
| (25R)-12alpha-hydroxy-24R,26R-dimethyl-26,27-cyclo-cholest-4-en-3,6-dione | LMST01100012 | C29H44O4 | 12.968021 | 0.0026666 | 6.1538992 |
| Soyasaponin III | HMDB0034651 | C42H68O14 | 12.667864 | 0.1708639 | -3.111586 |
| Voglibose | HMDB0015598 | C10H21NO7 | 12.494577 | 0.0144953 | 7.5182626 |
| Soyasaponin V | HMDB0039517 | C48H78O19 | 11.677737 | 0.1871888 | -5.397165 |
| 3alpha,15-Dihydroxymarasmene | HMDB0036039 | C15H22O4 | 11.641803 | 0.0435348 | -3.435916 |
| 3,5,6-Trihydroxy-5-(hydroxymethyl)-2-methoxy-2-cyclohexen-1-one | HMDB0041031 | C8H12O6 | 11.457023 | 0.0040449 | 4.574885 |
| 5-Acetamidovalerate | HMDB0012175 | C7H13NO3 | 11.124489 | 0.0084162 | 4.9208841 |
| (22S)-1alpha,22,25-trihydroxy-26,27-dimethyl-23,23,24,24-tetradehydrovitamin D3 / (22S)-1alpha,22,25-trihydroxy-26,27-dimethyl-23,23,24,24-tetradehydrocholecalciferol | LMST03020398 | C29H44O4 | 10.76079 | 0.0040185 | 4.5742329 |
| alpha-artemisic acid | 35491 | C18H32O3 | 10.459646 | 0.0142308 | -2.25265 |
| Blennin A | HMDB0031901 | C15H22O3 | 10.31254 | 0.0443266 | 1.1681404 |
| D-Glucuronic acid | HMDB0000127 | C6H10O7 | 9.9715431 | 0.0586312 | 1.4672893 |
| 2-(5,8-Tetradecadienyl)cyclobutanone | HMDB0037519 | C18H30O | 9.8347529 | 0.1927722 | 1.6245389 |
| 4-Acetyl-2(3H)-benzoxazolone | HMDB0040612 | C9H7NO3 | 9.6984733 | 0.0624606 | -5.193025 |
| Lysergic acid | 66676 | C16H16N2O2 | 9.0852802 | 0.0084129 | 5.6807811 |
| PS(O-16:0/0:0) | 78863 | C22H46NO8P | 8.6156429 | 0.00444 | -1.424974 |
| Ganoderic acid A | HMDB0034931 | C30H44O7 | 8.5800452 | 0.0861417 | 1.2416588 |
| Sandosapogenol | HMDB0031882 | C30H48O3 | 8.47296 | 0.1472765 | -6.110274 |
| Quillaic acid | LMPR0106150037 | C30H46O5 | 8.4044825 | 0.0044562 | 8.4284302 |
| Hydromorphone | HMDB0014472 | C17H19NO3 | 8.2378256 | 0.0332013 | 3.8113169 |
| 9(11)-Dehydroglycyrrhetic acid | HMDB0035251 | C30H46O3 | 8.0205751 | 0.0037396 | 4.8391984 |
| Candletoxin A | 67401 | C35H44O9 | 7.8700599 | 0.0067849 | 9.2708923 |
| (1beta,2alpha,3alpha)-1,2,3,24-Tetrahydroxy-12-oleanen-28-oic acid | HMDB0035487 | C30H48O6 | 7.8304601 | 0.0109242 | 1.7536291 |
| 28-Hydroxyglycyrrhetic acid | HMDB0035259 | C30H46O5 | 7.7276771 | 0.0171384 | 1.6553504 |
| PHORBOL MYRISTATE ACETATE | 44608 | C36H56O8 | 7.3731839 | 0.1962272 | -4.946337 |
| 12,13-Epoxy-9,15-octadecadienoic acid | 87400 | C18H30O3 | 7.2869543 | 0.0536291 | -1.344516 |
| Monotropein | HMDB0035608 | C16H22O11 | 7.2671595 | 0.0042735 | 6.1882243 |
| LysoPC(18:2(9Z,12Z)) | HMDB0010386 | C26H50NO7P | 7.1387267 | 0.1787862 | -2.437759 |
| 17-hydroxy-linolenic acid | LMFA02000239 | C18H30O3 | 7.0022624 | 0.0192698 | -2.081029 |
| Glabric acid | HMDB0034689 | C30H46O5 | 6.9926047 | 0.0016615 | 5.1839956 |
| octadeca-9Z,11E,14Z-trienoic acid | LMFA01030773 | C18H30O2 | 6.9581319 | 0.0134746 | -1.466879 |
| (±)-3',4'-Methylenedioxy-5,7-dimethylepicatechin | HMDB0037955 | C18H18O6 | 6.8641873 | 0.0771156 | -1.760785 |
| Difloxacin | 68932 | C21H19F2N3O3 | 6.6622212 | 0.0430614 | 3.5043683 |
| PC(14:0/20:1(11Z)) | HMDB0007879 | C42H82NO8P | 6.5990306 | 0.1234486 | -0.976826 |
| 1alpha,25-dihydroxy-26,27-dimethyl-22,22,23,23-tetradehydrovitamin D3 / 1alpha,25-dihydroxy-26,27-dimethyl-22,22,23,23-tetradehydro-cholecalciferol | LMST03020396 | C29H44O3 | 6.5638364 | 0.0020343 | 3.0336303 |
| D-Erythroascorbic acid 1'-a-D-xylopyranoside | HMDB0033626 | C10H14O9 | 6.3043495 | 0.008007 | 5.5975997 |
| N-Acetylneuraminic acid | HMDB0000230 | C11H19NO9 | 6.2564836 | 0.1823789 | 0.63188 |
| (3beta,17alpha,23S)-17,23-Epoxy-3,29-dihydroxy-27-norlanosta-7,9(11)-diene-15,24-dione | HMDB0035970 | C29H42O5 | 6.2154621 | 0.0133426 | 2.1149052 |
| Maprounic acid | LMPR0106180013 | C30H48O3 | 6.1278333 | 0.0025352 | 5.1247207 |
| (±)12,13-DiHOME | 45199 | C18H34O4 | 6.09468 | 0.0614242 | -1.484679 |
| Linusic acid | LMFA01050535 | C18H36O8 | 6.0908512 | 0.0004537 | -1.648917 |
| 4-Hydroxy-2-quinolone | 71293 | C9H7NO2 | 6.0354496 | 0.0037762 | -3.210931 |
| Cytochalasin Ppho | HMDB0035368 | C30H41NO6 | 5.859077 | 0.008897 | 9.3006604 |
| 2-O-(beta-D-galactopyranosyl-(1->6)-beta-D-galactopyranosyl) 2S-hydroxyundecanoic acid | LMFA13010047 | C23H42O13 | 5.8468055 | 0.0035677 | 7.6831948 |
| O-Desmethylangolensin | HMDB0004629 | C15H14O4 | 5.8240561 | 0.1823827 | -1.610464 |
| Medicagenic acid | HMDB0034551 | C30H46O6 | 5.7220551 | 0.0069942 | 4.0973023 |
| 6'-Hydroxyenterolactone | HMDB0041697 | C18H18O5 | 5.5934872 | 0.0569439 | 4.0511304 |
| (17alpha,23S)-Epoxy-28,29-dihydroxy-27-norlanost-8-ene-3,24-dione | HMDB0035624 | C29H44O5 | 5.5779371 | 0.0047126 | 2.808501 |
| 1-(O-alpha-D-glucopyranosyl)-3-keto-(1,27R,29R)-triacontanetriol | 46596 | C36H70O9 | 5.4357354 | 0.0183302 | -2.959709 |
| **Top 50 upregulated metabolites based on log_2_(FC) values** |  |  |  |  |  |
| Moschamine | HMDB0032759 | C20H20N2O4 | 4.3810014 | 0.0123016 | 50.513425 |
| PA(17:2(9Z,12Z)/22:6(4Z,7Z,10Z,13Z,16Z,19Z)) | LMGP10010305 | C42H67O8P | 2.3285781 | 0.0308679 | 48.929385 |
| Tetrahydrofolyl-[Glu](n) | HMDB0006826 | C29H37N9O12 | 1.9903084 | 0.0072 | 48.180866 |
| 26,27-diethyl-1alpha,25-dihydroxy-20,21-didehydro-23-oxavitamin D3/26,27-diethyl-1alpha,25-dihydroxy-20,21-didehydro-23- oxacholecalciferol | LMST03020462 | C30H48O4 | 1.9504938 | 0.0109009 | 48.13546 |
| PG(18:0/22:1(11Z)) | LMGP04010320 | C46H89O10P | 1.5646254 | 0.0170773 | 47.550334 |
| PPA(18:1(9Z)/18:1(9Z)) | LMGP11010002 | C39H74O11P2 | 1.2019296 | 0.0699305 | 47.266804 |
| PI(12:0/19:0) | LMGP06010025 | C40H77O13P | 1.3691525 | 0.0025341 | 47.015686 |
| Bis(methylsulfonylmethyl) disulfide | HMDB0041188 | C4H10O4S4 | 1.1451847 | 0.0036654 | 46.544059 |
| (Z,Z)-2,9,16-Heptadecatriene-4,6-diyn-8-ol | HMDB0032674 | C17H22O | 1.6519161 | 0.0027193 | 14.945011 |
| Chlorin E6 | HMDB0059948 | C34H36N4O6 | 5.2537743 | 0.0025355 | 13.713325 |
| 5,4'-Dihydroxy-3,8,3'-trimethoxy-7-prenyloxyflavone | LMPK12113223 | C23H24O8 | 1.36998 | 0.1651056 | 12.776369 |
| Deoxyhypusine | HMDB0011150 | C10H23N3O2 | 3.6841134 | 0.0119123 | 12.687788 |
| 3,4,5-trihydroxy-6-[(14-hydroxy-3-methyl-1,7-dioxo-3,4,5,6,7,8,9,10-octahydro-1H-2-benzoxacyclotetradecin-16-yl)oxy]oxane-2-carboxylic acid | HMDB0134196 | C24H30O11 | 2.3994588 | 0.0095416 | 11.5873 |
| Idarubicin | HMDB0015308 | C26H27NO9 | 1.0469329 | 0.1154706 | 11.44213 |
| 3-O-cis-Coumaroylmaslinic acid | HMDB0034539 | C39H54O6 | 1.0447695 | 0.006993 | 11.264602 |
| Ascladiol | HMDB0029610 | C7H8O4 | 1.02562 | 0.0043182 | 10.753655 |
| Thonzylamine | HMDB0240222 | C16H22N4O | 2.0133399 | 0.1108661 | 10.218689 |
| Moracin I | HMDB0033311 | C20H20O4 | 3.0204908 | 0.0348181 | 9.745023 |
| 6-s-cis-locked retinal | LMPR01090039 | C20H26O | 3.9702467 | 0.0056728 | 9.4840079 |
| PC(DiMe(11,3)/DiMe(11,5)) | HMDB0061391 | C50H89NO10P+ | 1.4605273 | 0.0192487 | 9.4640101 |
| Diltiazem | HMDB0014487 | C22H26N2O4S | 1.1250803 | 0.0039023 | 9.4180613 |
| Neferine | HMDB0034104 | C38H44N2O6 | 1.8299252 | 0.0563652 | 9.4031965 |
| Prostaglandin D2-biotin | 44944 | C36H60N4O6S | 2.3658979 | 0.0121799 | 9.3958138 |
| Cytochalasin Ppho | HMDB0035368 | C30H41NO6 | 5.859077 | 0.008897 | 9.3006604 |
| Candletoxin A | 67401 | C35H44O9 | 7.8700599 | 0.0067849 | 9.2708923 |
| Formyl-5-hydroxykynurenamine | HMDB0012948 | C10H12N2O3 | 2.2149083 | 0.0098536 | 8.9557394 |
| 18-Dehydroursolic acid 3-arabinoside | HMDB0037908 | C35H54O7 | 3.3009382 | 0.0061836 | 8.7973681 |
| 1beta,3beta,5alpha,6beta-tetrahydroxyandrostan-17-one | LMST02020108 | C19H30O5 | 3.4403442 | 0.0039861 | 8.6427111 |
| 6-O-(3R,4-dihydroxy-2-methylene-butanoyl)-beta-D-glucopyranose | LMSL05000003 | C11H18O9 | 3.4836529 | 0.0032022 | 8.5280303 |
| N-stearoyl taurine | LMFA08020078 | C20H41NO4S | 1.7339458 | 0.003877 | 8.5152989 |
| Ganoderic acid DM | HMDB0032837 | C30H44O4 | 1.0797066 | 0.0025157 | 8.4625761 |
| PG(O-16:0/14:1(9Z)) | LMGP04020004 | C36H71O9P | 1.2143512 | 0.0064132 | 8.457774 |
| ponasterone A | LMST01010195 | C27H44O6 | 2.9699739 | 0.029665 | 8.4510874 |
| Quillaic acid | LMPR0106150037 | C30H46O5 | 8.4044825 | 0.0044562 | 8.4284302 |
| 6-(2,4-dihydroxyphenyl)-2-(2,6-dihydroxyphenyl)-5-hydroxy-4-methylcyclohex-3-ene-1-carboxylic acid | HMDB0126474 | C20H20O7 | 1.0636233 | 0.0124941 | 8.4060786 |
| Veranisatin C | HMDB0031756 | C16H20O10 | 3.9572891 | 0.0038649 | 8.3943164 |
| [(3-methylbut-2-en-1-yl)oxy]sulfonic acid | HMDB0136691 | C5H10O4S | 2.1109277 | 0.0078664 | 8.236126 |
| Teinemine | LMST01150010 | C27H45NO2 | 1.6532691 | 0.0266072 | 8.1347848 |
| Vitexin | 45737 | C21H20O10 | 1.9445165 | 0.0878934 | 8.0998969 |
| 3-trans-p-Coumaroylrotundic acid | HMDB0040667 | C39H54O7 | 1.2069669 | 0.0064719 | 8.0175232 |
| 3,4,5-trihydroxy-6-(2-oxoethoxy)oxane-2-carboxylic acid | HMDB0142000 | C8H12O8 | 3.7203163 | 0.0122564 | 7.9564242 |
| 2-Arachidonoylglycerophosphocholine | HMDB0061699 | C28H51NO7P+ | 2.3765006 | 0.0031867 | 7.7053974 |
| Artonol C | HMDB0030489 | C30H28O7 | 1.6718622 | 0.0069778 | 7.6931999 |
| 2-O-(beta-D-galactopyranosyl-(1->6)-beta-D-galactopyranosyl) 2S-hydroxyundecanoic acid | LMFA13010047 | C23H42O13 | 5.8468055 | 0.0035677 | 7.6831948 |
| Edetic Acid | HMDB0015109 | C10H16N2O8 | 1.1780184 | 0.0077912 | 7.6310234 |
| Pratensin B | LMPK12113304 | C23H24O9 | 1.17821 | 0.1241047 | 7.6300128 |
| Voglibose | HMDB0015598 | C10H21NO7 | 12.494577 | 0.0144953 | 7.5182626 |
| [2-hydroxy-5-(3,5,7-trihydroxy-3,4-dihydro-2H-1-benzopyran-2-yl)phenyl]oxidanesulfonic acid | HMDB0127729 | C15H14O9S | 1.0526234 | 0.134367 | 7.3268626 |
| Zeanic acid | HMDB0032963 | C10H7NO4 | 1.5098662 | 0.0249591 | 7.313077 |
| Episterol | HMDB0006847 | C28H46O | 1.2753506 | 0.041948 | 7.2974846 |
| **Top 50 downregulated metabolites based on log_2_(FC) values** |  |  |  |  |  |
| 4'-O-Methylbavachalcone | LMPK12120047 | C22H24O4 | 2.1707506 | 0.1603861 | -10.89826 |
| Saponin H | HMDB0035607 | C36H58O10 | 1.132837 | 0.1710247 | -8.317534 |
| (E)-5-(hexa-3,5-dien-1-yn-1-yl)-5'-(prop-1-yn-1-yl)-2,2'-bithiophene | LMFA12000358 | C17H12S2 | 1.3184009 | 0.0573312 | -8.011273 |
| 23-trans-p-Coumaroyloxytormentic acid | HMDB0040682 | C39H54O8 | 2.6381648 | 0.1564747 | -7.457382 |
| 3-[4-Hydroxy-3-(3-methyl-2-butenyl)phenyl]-2-propenal | HMDB0040833 | C14H16O2 | 1.0988485 | 0.1925693 | -7.003907 |
| Sulfamerazine | HMDB0015521 | C11H12N4O2S | 1.2520721 | 0.033503 | -6.978057 |
| Propinol adenylate | HMDB0006806 | C13H18N5O8P | 1.0644012 | 0.0716869 | -6.973965 |
| Chondrillasterol 3-[glucosyl-(1->4)-glucoside] | HMDB0033886 | C41H68O11 | 2.3851214 | 0.1969004 | -6.797339 |
| Synaptolepis factor K1 | 67502 | C36H54O8 | 2.888779 | 0.094892 | -6.677462 |
| Ethylsuberenol | HMDB0039044 | C17H20O4 | 2.0743966 | 0.1678755 | -6.511688 |
| 2,4-Dihydroxychalcone | HMDB0039612 | C15H12O3 | 1.1928542 | 0.0482411 | -6.460572 |
| Dehydrosoyasaponin I | HMDB0039331 | C48H76O18 | 2.4754488 | 0.0768385 | -6.45813 |
| Madlongiside D | HMDB0037905 | C41H66O14 | 1.6408899 | 0.1008506 | -6.330866 |
| Isochamanetin | LMPK12140146 | C22H18O5 | 1.9534382 | 0.177242 | -6.305217 |
| (R)-2-Hydroxy-7,8-dimethoxy-2H-1,4-benzoxazin-3(4H)-one 2-glucoside | HMDB0037262 | C16H21NO10 | 1.7445954 | 0.0273972 | -6.301872 |
| Kukoamine D | HMDB0060527 | C28H42N4O6 | 1.6655147 | 0.0726015 | -6.134029 |
| Sandosapogenol | HMDB0031882 | C30H48O3 | 8.47296 | 0.1472765 | -6.110274 |
| Asiaticoside B | HMDB0029892 | C48H78O20 | 2.8692313 | 0.1144572 | -6.105854 |
| Acutoside A | HMDB0031021 | C42H68O13 | 3.5998601 | 0.1967226 | -6.073492 |
| N-Malonylanthranilate | 65916 | C10H9NO5 | 3.1874583 | 0.062045 | -5.830106 |
| Imatinib | HMDB0014757 | C29H31N7O | 1.6722087 | 0.0479617 | -5.57936 |
| Lercanidipine | HMDB0014669 | C36H41N3O6 | 5.1781587 | 0.092365 | -5.434212 |
| 3alpha-3-Hydroxytirucalla-7,24-dien-21-oic acid | HMDB0034962 | C30H48O3 | 4.1433867 | 0.1045346 | -5.420992 |
| Iprobenfos | HMDB0031768 | C13H21O3PS | 2.9102041 | 0.1539735 | -5.413439 |
| Soyasaponin V | HMDB0039517 | C48H78O19 | 11.677737 | 0.1871888 | -5.397165 |
| 4-Acetyl-2(3H)-benzoxazolone | HMDB0040612 | C9H7NO3 | 9.6984733 | 0.0624606 | -5.193025 |
| [2-hydroxy-3-(phenoxycarbonyl)phenyl]oxidanesulfonic acid | HMDB0135171 | C13H10O7S | 1.0823956 | 0.1478497 | -5.054414 |
| 1-Heptadecanoylglycerophosphoethanolamine | HMDB0061691 | C22H46NO7P | 2.3213298 | 0.1152224 | -5.010871 |
| PHORBOL MYRISTATE ACETATE | 44608 | C36H56O8 | 7.3731839 | 0.1962272 | -4.946337 |
| Tazarotenic acid | HMDB0060619 | C19H17NO2S | 2.8432925 | 0.0704183 | -4.752403 |
| Calenduloside B | HMDB0039413 | C48H78O18 | 15.997019 | 0.1737437 | -4.662279 |
| Kinetensin 4-7 | HMDB0012986 | C26H37N9O6 | 1.4374633 | 0.1074087 | -4.640156 |
| 3'-demethyletoposide | HMDB0061028 | C28H30O13 | 1.1332305 | 0.1403823 | -4.628093 |
| OKOHA-PE | LMGP20020040 | C30H52NO10P | 1.270276 | 0.0114402 | -4.618718 |
| 2-[4-(3-Hydroxypropyl)-2-methoxyphenoxy]-1,3-propanediol | HMDB0040352 | C13H20O5 | 2.3528852 | 0.1360702 | -4.585125 |
| Xanthurenic acid | 5841 | C10H7NO4 | 1.0539503 | 0.0046339 | -4.570244 |
| repaglinide aromatic amine | HMDB0061005 | C22H28N2O4 | 2.575773 | 0.0563652 | -4.529527 |
| Momordin I | HMDB0036358 | C41H64O13 | 1.0458554 | 0.1631025 | -4.419248 |
| Aflatoxin B1 dialcohol | HMDB0011672 | C18H18O6 | 1.9232853 | 0.0948337 | -4.279044 |
| Chondroitin 4-sulfate | HMDB0000652 | C14H23NO15S | 1.3498731 | 0.1805938 | -4.198827 |
| Cinncassiol D2 glucoside | HMDB0034679 | C26H42O11 | 4.1079124 | 0.0880719 | -4.164668 |
| N-decanoyl-L-Homoserine lactone | 45310 | C14H25NO3 | 2.3617679 | 0.0380567 | -4.125779 |
| Indole-3-carboxilic acid-O-sulphate | HMDB0060002 | C9H7NO5S | 5.0437359 | 0.1659811 | -4.055981 |
| Withaperuvin C | 89699 | C28H38O7 | 1.4230388 | 0.0283883 | -4.024735 |
| Galbanic acid | HMDB0030163 | C24H30O5 | 2.6726612 | 0.0050541 | -3.977049 |
| cis-Resveratrol 3-sulfate | HMDB0041712 | C14H12O6S | 1.7607753 | 0.0961981 | -3.972461 |
| E-10-Hydroxydesmethylnortriptyline | HMDB0014257 | C18H19NO | 1.2147123 | 0.0326697 | -3.956625 |
| Polysorbate 60 | HMDB0037183 | C22H42O8 | 1.0365677 | 0.0173806 | -3.868543 |
| Ansamitocin P3 | LMPK04000039 | C32H43ClN2O9 | 1.3760634 | 0.1193447 | -3.854266 |
| N-arachidonoyl taurine | LMFA08020075 | C22H37NO4S | 3.1043624 | 0.1909781 | -3.853657 |
