# Supplementary material for: Anti-aging effects of a functional food via the action of gut microbiota and metabolites in aging mice
Source: Aging (Albany NY). 2021 Apr 20;13(13):17880–900. doi: 10.18632/aging.202873 (PMC8312451; doi:10.18632/aging.202873)
Supplement: Supplementary Table 4 [file aging-13-202873-s004.pdf]

Supplementary Table 4. The top 50 differentially metabolites based on VIP values identified between WS and SD groups from serum samples.

Metabolites	Compound ID	Formula	VIP	adj.P-value	log2(FC)
PA(17:1(9Z)/22:2(13Z,16Z))	LMGP10010273	C42H77O8P	97.314967	0.209804	-0.895337
LysoPC(18:1(11Z))	HMDB0010385	C26H52NO7P	18.311347	0.3165841	-0.34309
PC(14:0/22:4(7Z,10Z,13Z,16Z))	HMDB0007889	C44H80NO8P	14.032345	0.2252829	0.649401
PC(16:0/20:5(5Z,8Z,11Z,14Z,17Z))	HMDB0007984	C44H78NO8P	11.516296	0.1951097	-0.955838
PE(19:0/20:2(11Z,14Z))	LMGP02010783	C44H84NO8P	10.620062	0.1921897	0.7109557
LysoPC(20:1(11Z))	HMDB0010391	C28H56NO7P	10.48363	0.1212164	-0.722421
PE-NMe2(18:0/20:4(5Z,8Z,11Z,14Z))	HMDB0113999	C45H82NO8P	10.160781	0.2333785	-1.244559
LysoPC(18:1(9Z))	HMDB0002815	C26H52NO7P	9.0009403	0.2231003	-0.539019
L-Isoleucine	23	C6H13NO2	8.9537036	0.0824465	-0.554532
LysoPC(20:5(5Z,8Z,11Z,14Z,17Z))	HMDB0010397	C28H48NO7P	8.6833586	0.1888057	-1.704422
PC(16:0/20:4(5Z,8Z,10E,14Z)(12OH[S]))	LMGP20010002	C44H80NO9P	7.8218772	0.1745241	-0.929309
Dihydrocoumarin	65745	C9H8O2	7.4571693	0.1189771	-0.492561
LysoPC(P-18:0)	HMDB0013122	C26H54NO6P	7.4562366	0.0474968	0.7093557
PC(16:0/18:3(9Z,12Z,15Z))	HMDB0007975	C42H78NO8P	7.0461573	0.0307741	1.4723047
PE-NMe(16:0/22:4(7Z,10Z,13Z,16Z))	HMDB0113055	C44H80NO8P	6.9274977	0.0829941	-1.494256
LysoPC(16:1(9Z)/0:0)	HMDB0010383	C24H48NO7P	6.6465147	0.1030072	-0.947832
PC(18:0/22:6(4Z,7Z,10Z,13Z,16Z,19Z))	HMDB0008057	C48H84NO8P	5.7353409	0.2631859	-0.825004
[4-(7-hydroxy-3,4-dihydro-2H-1-benzopyran-3-yl)phenyl]oxidanesulfonic acid	HMDB0141213	C15H14O6S	5.5775041	0.0012168	-2.359003
PC(16:0/16:1(9Z))	HMDB0007969	C40H78NO8P	5.1757936	0.3061426	-0.93991
LysoPC(18:3(6Z,9Z,12Z))	HMDB0010387	C26H48NO7P	5.1445447	0.2939349	1.0303031
PC(18:3(6Z,9Z,12Z)/P-16:0)	HMDB0008192	C42H78NO7P	4.8624727	0.0309988	4.0621769
SM(d16:1/17:0)	LMSP03010037	C38H77N2O6P	4.8170665	0.175073	1.0270691
PS(P-18:0/0:0)	LMGP03070002	C24H48NO8P	4.6128137	0.2902771	1.7678306
PC(19:1(9Z)/0:0)	LMGP01050130	C27H54NO7P	4.4287531	0.0507577	-0.998555
2-Hydroxycinnamic acid	306	C9H8O3	4.3767768	0.1862954	-0.3715
PS(O-18:0/0:0)	LMGP03060002	C24H50NO8P	4.2481394	0.2269156	-2.151201
PE(20:3(5Z,8Z,11Z)/22:6(4Z,7Z,10Z,13Z,16Z,19Z))	HMDB0009342	C47H76NO8P	3.7751669	0.1696274	-1.398126
PC(O-18:1(11Z)/0:0)	LMGP01060034	C26H54NO6P	3.525407	0.0056784	1.7496163
PS(18:2(9Z,12Z)/0:0)	LMGP03050011	C24H44NO9P	3.4711804	0.0006819	4.0646739
PC(O-20:0/0:0)	LMGP01060041	C28H60NO6P	3.1728602	0.1396623	1.0487906
2-amino-6-hydroxyhexanoic acid	HMDB0142963	C6H13NO3	3.0204929	0.0032674	1.1836564
PC(16:0/20:4(5Z,8Z,11Z,14Z))	HMDB0007982	C44H80NO8P	2.9742859	0.062752	-1.379219
5β-CHOLANIC ACID-3α, 12α-DIOL N-(2-SULPHOETHYL)-AMIDE	44688	C26H45NO6S	2.9194186	0.0024679	3.4147921
Glu Leu	44684	C11H20N2O5	2.7882774	0.1058725	-0.790463
10,11-dihydro-20-trihydroxy-leukotriene B4	HMDB0012503	C20H34O7	2.7657274	0.2583607	1.3817179
PE(19:0/20:4(5Z,8Z,11Z,14Z))	LMGP02010785	C44H80NO8P	2.735602	0.0588195	-1.447664
2-O-methyl PAF C-16	43417	C25H54NO6P	2.4993142	0.0039339	2.6908699
2-Heptanethiol	HMDB0032303	C7H16S	2.4603179	0.0845367	-0.562484
LysoPE(0:0/20:5(5Z,8Z,11Z,14Z,17Z))	HMDB0011489	C25H42NO7P	2.455469	0.0192129	-1.376139
Cholesteryl-alpha-D-glucoside	LMST05050029	C32H54O6	2.3441144	0.0461908	-5.753648
{4-[(1E)-3-(4-hydroxy-2-methoxyphenyl)prop-1-en-1-yl]phenyl}oxidanesulfonic acid	HMDB0130353	C16H16O6S	2.3387177	0.0083457	-2.38248
3-(4-Methyl-3-pentenyl)thiophene	92919	C10H14S	2.3260856	0.1212665	-0.491184
PE(P-18:0/20:4(6E,8Z,11Z,14Z)(5OH[S]))	82394	C43H78NO8P	2.2653099	0.2514598	1.0801764
THTC	68817	C5H8O2S	2.2653005	0.1299283	-0.365755
SM C16:1	HMDB0029216	C40H80N2O6P+	2.228824	0.2024559	1.1942671
17-hydroxy stearic acid	LMFA02000135	C18H36O3	2.1478087	0.1745841	-0.473623
L-Carnitine	34532	C7H15NO3	2.1361484	0.2269156	0.290201
PC(21:0/0:0)	40317	C29H60NO7P	2.1260332	0.1819852	1.0141365
Equol 4'-O-glucuronide	HMDB0041731	C21H22O9	2.0921303	0.003526	-2.94973
L-Proline	29	C5H9NO2	2.0847472	0.2193284	-0.462759
